# Investigation of intermediate CAG alleles of the *HTT* in the general population of Rio de Janeiro, Brazil, in comparison with a sample of Huntington disease‐affected families

**DOI:** 10.1002/mgg3.1181

**Published:** 2020-02-17

**Authors:** Thays A. Apolinário, Iane dos Santos da Silva, Luciana de Andrade Agostinho, Carmen L. A. Paiva

**Affiliations:** ^1^ Programa de Pós‐Graduação em Neurologia Universidade Federal do Estado do Rio de Janeiro (UNIRIO) Rio de Janeiro RJ Brazil; ^2^ Programa de Pós‐Graduação em Biologia Molecular e Celular Universidade Federal do Estado do Rio de Janeiro (UNIRIO) Rio de Janeiro RJ Brazil; ^3^ Centro Universitário FAMINAS – UNIFAMINAS Muriaé MG Brazil; ^4^ Fundação Cristiano Varella‐Hospital do Câncer Muriaé MG Brazil

**Keywords:** Brazilian sample, *HTT*, Huntington disease, intermediate alleles

## Abstract

**Background:**

Huntington disease (HD) (MIM: 143100) is a severe autosomal dominant neurodegenerative disease caused by the expansion of CAG trinucleotides (>35) in the *HTT*.

**Objective:**

To investigate the frequency of intermediate CAG alleles (IAs) in individuals residing in Rio de Janeiro city with no familial history of HD (general population, GP) in comparison with a sample of individuals from families presenting with HD who were previously investigated by our group (affected sample, AS).

**Results:**

The frequency of normal CAG alleles was 96.2%, while that of IAs was 3.6%, and that of reduced penetrance alleles was 0.2% in the GP (*n* = 470 chromosomes); 7.2% (17/235 individuals) of the GP presented an IA in heterozygosis with a normal allele. There was no statistically significant difference between the frequencies of the IAs in the GP and in the AS (*p* = .9). The most frequent haplotype per normal allele was (CAG)17‐(CCG)7 (101/461) and per IA was (CAG)27‐(CCG)7 (6/17) in the GP. These haplotypes were also the most frequent in the normal and IA chromosomes of the AS, respectively.

**Conclusion:**

The genetic profiles of the IAs obtained from GP and AS were rather similar. It is important to investigate the frequencies of the IAs because expansions arise from a step‐by‐step mechanism in which, during intergenerational transmission, large normal alleles can generate IAs, which are then responsible for generating de novo HD mutations. The genetic investigation of IAs in the GP was also important because it was focused on the population of Rio de Janeiro, an understudied group. CCG7 was the most frequent CCG allele in linkage disequilibrium with normal, intermediate, and expanded CAG alleles, similar to the Western Europe population. However, a more robust investigation, in conjunction with haplogroup determination (A, B, or C), will be required to elucidate the ancestral origin of the *HTT* mutations in Brazilians.

## Graphical abstract

The genetic profiles of CAG and CCG polymorphic regions were compared between the general and affected samples, as was the frequency of intermediate alleles (IAs). In the general population, the frequency of IAs was 3.6% (*n* = 470 chromosomes). There was no statistically significant difference between the frequency of IAs in the general population and the frequency of IAs in the affected sample.

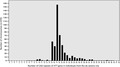

## 1 INTRODUCTION

Huntington disease (HD) (MIM: 143100) is a severe autosomal dominant neurodegenerative disease caused by the expansion of CAG trinucleotides in the *HTT* (4p16.3) above the normal threshold (>35). The onset of HD symptoms usually occurs in the fourth decade of life. As a result of the expansion of CAG repeats, the protein encoded by this mutated gene, mutated Huntingtin (mHTT), is expressed with a longer than normal polyglutamine tail (Hayden 1981 apud Gusella et al., 1983; Gusella & MacDonald, 1996; The Huntington Disease Research Group, 1993).

Alleles considered normal exhibit fewer than 27 CAG copies, leading to an abnormal phenotype (HDCRG 1993; Duyao et al. 1993; Benjamin et al. 1994; Kremer et al. 1994 apud The American College of Medical Genetics/American Society of Human Genetics Huntington Disease Genetic Testing Working Group., 1998). Intermediate alleles (IAs) (27–35 CAGs) are genetically unstable during mitosis and meiosis and may favor CAG expansions over generations. Because they are genetically unstable, IAs present a risk of generating de novo HD mutations. Therefore, it can be suggested that *HTT* expansions are replenished in a population due to the pool of IAs present in that population (Kremer et al. 1994; Rubinsztein et al. 1996; Leeflang et al. 1995; Telenius et al. 1995; Chong et al. 1997 apud The American College of Medical Genetics/American Society of Human Genetics Huntington Disease Genetic Testing Working Group., 1998; Kay et al., 2018). Alleles with 36–39 CAG repeats are referred to as alleles with reduced penetrance and may or may not generate HD phenotypes in people who carry them (Legius et al. 1994; Rubinsztein et al. 1996; McNeil et al. 1997; U.S. HD Genetic Testing Group, unpublished data apud The American College of Medical Genetics/American Society of Human Genetics Huntington Disease Genetic Testing Working Group., 1998). Alleles with more than 39 CAG copies show full penetrance and inexorably cause the disease (The American College of Medical Genetics/American Society of Human Genetics Huntington Disease Genetic Testing Working Group., 1998).

In contrast to what has been believed about the phenotypes of patients with IAs, a longitudinal study conducted in a group of individuals bearing IAs suggested the existence of phenotypic behavioral changes (apathy and a tendency toward suicide) and mild cognitive and motor changes (Killoran et al., 2013). The association of these alleles with the presence of clinical signs means that the bearers of IAs present clinical relevance. These results have important implications not only for the pathogenesis of the disease but also for genetic counseling, since in most cases, these individuals receive genetic counseling with the guarantee that they are asymptomatic (Feigin et al., 2006; Semaka, Kay, Doty, Collins, Tam, et al., 2013; Semaka, Kay, Doty, Collins, Bijlsma, et al., 2013).

Near the polymorphic CAG region in the *HTT*, only 12 bp from the CAG region, there is another polymorphic region of CCG repeats that needs to be investigated because this CCG region has been used for genetic studies related to the ancestral origin of the mutated *HTT* (The Huntington disease Research group, 1993). The main objective of this study was to investigate the frequency of IAs in the *HTT* genes of individuals residing in Rio de Janeiro city who show no familial history of HD (general population, GP) in comparison with a sample of individuals from HD‐affected families (affected sample, AS) previously investigated by our group (Agostinho, 2011; Agostinho et al., 2012, 2015). To this end, we sized CAG and CCG repeats both independently and in phase (CAG/CCG haplotypes) in the *HTT*.

## 2 INDIVIDUALS AND METHODS

### 2.1 Ethical compliance

The project was approved by the Ethics in Research Committee (REC) of the Hospital Universitário Gaffree e Guinle/UNIRIO (HUGG) (CAAE number 51547615.2.0000.5258 and CAAE number 26387113.1.0000.5258). The samples were made anonymous to avoid unnecessary stress to individuals who presented with intermediate or reduced penetrance alleles. The subjects consented to participate in the study only as donors of anonymous samples.

### 2.2 Sample size calculation

The sample size was calculated in line with the Netquest Sample Calculator (https://www.netquest.com/calculate-your-sample-size) as follows. Size of the universe: Number of total inhabitants of Rio de Janeiro as calculated by IBGE in 2016:6,498,837; number of chromosomes: 12,997,674; 50% heterogeneity; margin of error 5%; and confidence level 95%. Calculated sample size: *n* = 385 chromosomes. Sample size used: *n* = 470 chromosomes.

### 2.3 Subject recruitment

Unrelated individuals were randomly selected among students from the Universidade Federal do Estado do Rio de Janeiro (UNIRIO) who were aged 18 years and older without a family history of HD (GP). Related individuals from HD‐affected families who were also residents of Rio de Janeiro city (molecularly positive and symptomatic or asymptomatic as well as molecularly negative) were recruited from Agostinho's (2011) database as the AS.

### 2.4 Molecular investigation

Peripheral blood was collected from the individuals for subsequent DNA extraction (with a Mini Prep Spin Kit, GE Healthcare UK Limited, Little Chalfont, UK). To determine the allelic profiles of the affected and unaffected individuals, the numbers of repeats of the CAG and CCG alleles of the *HTT* [Genomic Coordinates—(GRCh38): 4:3,074,680‐3,243,959] were determined according to the technique described by Agostinho (2011) and validated by Agostinho et al. (2015), adding a Sanger sequencing validation step using different sizes of CAG alleles as positive controls.

CAG and CAG‐CCG analysis: Three primers were used to amplify the target DNA sequences (CAG and CAG/CCG polymorphic regions). For CAG repeats only, 6 FAM 5′‐TGGCGACCCTGGAAAAGCTGAT‐3′ (forward 1, HD1) and 5′‐GCGGTGGCGGCTGTTGCTGCT‐3′ (reverse 1, HD3) were used. For CAG‐CCG repeats, we used the forward 1 (HD1) and 5′‐CGG CGGCGGCTGAGGAAGCTG‐3′ (reverse 2, HD4) primers.

The PCR mix for each reaction contained 0–100 ng of DNA, 10 pmols of each primer, 6.25 µl of Promega GoTaq Green Master Mix (containing 1.5‐mM MgCl_2_ and each dNTP at 200 mM), and sufficient DNase‐free water to obtain a final volume of 12.5 µl. Two distinct products were PCR amplified; one of the products was used to analyze the polymorphic CAG repeat region, and the other product was used to analyze both the CAG and CCG repeat regions in phase. The thermal cycling conditions were 94°C for 5 min; 35 cycles of 94°C for 1 min, 59.1°C for 1 min, and 72°C for 2 min; and a single cycle of 72°C for 50 min. Allele profiles were determined on an ABI 3,500 Genetic Analyzer (Applied Biosystems). The data were analyzed using the GeneScan Analysis 3.7 and Genotyper 3.7 programs (Applied Biosystems).

To genotype the CCG polymorphic region alone and, thus, validate the above CAG‐CCG analysis, the following pair of primers was used: 6 FAM—5′AACAGCCGCCACCGCCGC‐3′ (forward 2, D4S3076662F) and 5′‐GCGGCTGAGGCAGCAGCGG‐3′ (reverse 3, D4S3076748R). Each amplification reaction contained 2 ng of DNA, 10 pmols of each primer, 6.25 µl of Master Mix (containing 1.0‐mM MgCl_2_, and each dNTP at 200 mM) (Invitrogen, Life Technologies), and sufficient DNase‐free water to reach a final volume of 12.5 µl. The thermal cycling conditions were as follows: 94°C for 5 min; 35 cycles of 94°C for 1 min and 72°C for 3 min (the same temperature was used for annealing and elongation).

### 2.5 Statistical analysis

Data were statistically analyzed with SPSS version 17. A descriptive analysis was performed to calculate the means, medians, standard deviations, and frequencies. The Wilcoxon posttest and the Chi‐square test with the correction of Yates were used to compare IA frequencies in the GP and AS. Relative risks (odds ratio with the Haldane correction) were calculated when the difference between the frequencies was statistically significant.

## 3 RESULTS

**Figure 1:**
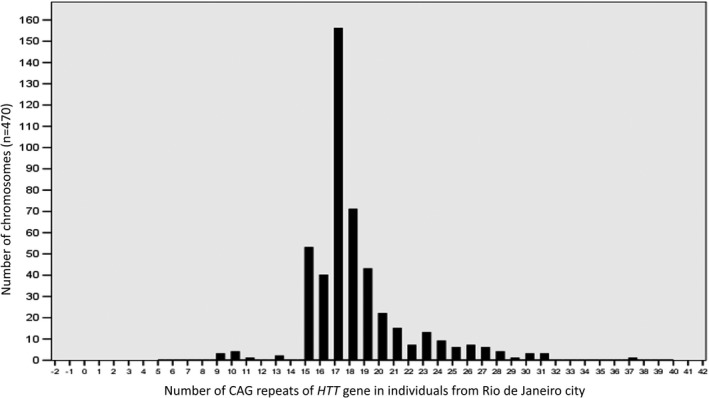
Number of the CAG repeats of the HTT in individuals from Rio de Janeiro city

We investigated 478 chromosomes of 239 individuals from the GP. CAG alleles were identified in 235 individuals (470 chromosomes) from this group. For normal CAG alleles (*n* = 452), the mean number of repeats was 17.8 (±2.6) and the median was 17 (ranging from 9 to 26). Seventeen IAs were identified among 470 total chromosomes (3.6%) corresponding to 17/235 heterozygous individuals (7.23%). Among these 17 IAs, the following numbers of CAG repeats were found: 27 (*n* = 6), 28 (*n* = 4), 29 (*n* = 1), 30 (*n* = 3), and 31 (*n* = 3). One individual exhibited an allele with reduced penetrance (0.2%) with 37 CAG repeats. The absolute frequencies of the CAG repeats in the chromosomes from the GP are shown in Figure 1.

**Figure 2:**
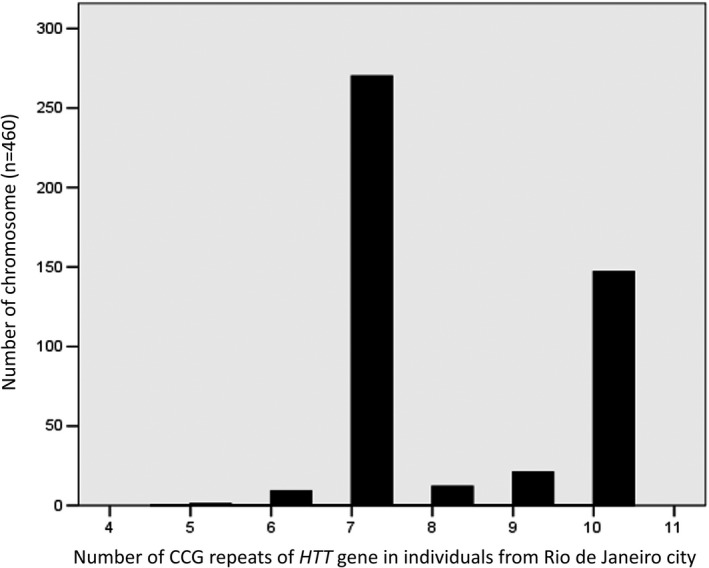
Number of the CCG repeats of the HTT in individuals from Rio de Janeiro city

CCG alleles were analyzed in 230/239 individuals from the GP (Figure 2). The number of CCG repeats among the 460 alleles ranged from 5 to 10, with a median of 7. CCG7 alleles were found in 255 chromosomes (55.43%), and 145 chromosomes harbored CCG10 (31.52%).

CAG repeats in phase with CCG repeats were categorized as the minor and major CAG alleles per individual (Table S1).

The most frequent haplotype per normal allele in the GP was (CAG)17‐(CCG)7, and the most frequent haplotype per IA in the same population was (CAG)27‐(CCG)7. The former haplotype exhibited an absolute frequency of 101 and a relative frequency of 0.208 (*n* = 461 chromosomes), and the latter exhibited an absolute frequency of 6 and a relative frequency of 0.352 (*n* = 17 chromosomes) (Table S2). On the other hand, Tables S3 and S4 show the frequencies of CAG‐CCG haplotypes in the sample of HD‐affected individuals. Haplotypes (CAG)17‐(CCG)7 and (CAG)27‐(CCG)7 were also the most frequent among the normal and IA alleles in the AS (Tables S3 and S4).

In the sample of individuals from affected HD families, 110 chromosomes from 55 individuals were analyzed: 36/55 individuals exhibited one expanded allele each (>39 CAG repeats). Among these 36 individuals, three were heterozygous with one expanded allele and one IA, and 33 were heterozygous with an expanded allele and a normal allele, while 18/55 individuals exhibited two normal alleles, and one individual was heterozygous with one normal allele and one IA. The mean number of CAG repeats in the normal alleles of this group of affected families was 18.2 (±2.9) and the median was 17; in addition, the normal CAG allele with the lowest number of repeats contained 14 CAG repeats, and that with the greatest number contained 26 repeats. The frequency of IAs among the chromosomes of the AS was 4/110, corresponding to 3.62% of chromosomes or 4/55 individuals (7.27%). Among the four IA carriers from the AF, two exhibited 27 CAG repeats, while one exhibited 28, and the other exhibited 29. The mean number of expanded CAG repeats in the affected group was 46.36 ± 6.5.

In relation to the CCG polymorphic region, 81 chromosomes from the AS exhibited CCG7 alleles (75%), and 21 chromosomes presented CCG10 (19.4%). One individual who was not subjected to CCG analysis was excluded. The most frequent type of CCG allele in linkage disequilibrium with IAs was CCG7 (*n* = 4). There was no statistically significant difference between the frequencies of the IAs in the AS and the GP (*X* ^2^ = 0.03, odds ratio = 1.0, and *p* = .9).

The alleles of both samples were categorized as normal, intermediate, reduced penetrance, or full penetrance alleles (Table S5). The normal CAG alleles of individuals from the GP were compared with the normal‐sized CAG alleles of HD carriers. In this comparison, no statistically significant difference was observed (*p* = 1).

When the frequency of CCG10 alleles in phase with normal CAG alleles from the GP was compared with the frequency of CCG10 alleles in phase with expanded CAG alleles, it was observed that the occurrence of these alleles in the normal chromosomes was 12.7 times higher than that in chromosomes bearing expanded CAG alleles (AS) (*χ* ^2^ = 12, odds ratio 12.7, and *p* = .0005).

## 4 DISCUSSION

The frequency of IAs ranges from 1.0% to 3.9% in the GP of North American countries (Semaka et al., 2006; The Huntington Disease Research Group, 1993). According to Raskin et al. (2000), in Brazil, the frequency of IAs found among 50 chromosomes from the GP of Paraná State was 8.7% (Raskin et al., 2000). In the present study, the frequency of IAs in the GP of Rio de Janeiro city was 7.23% (*n* = 235 individuals). These frequencies of IAs found in Brazil, a country with an admixed population, are higher than those in the GP of Western Europe (5.8%; *n* = 1,594 individuals) (Semaka, Kay, Doty, Collins, Tam, et al., 2013; Semaka, Kay, Doty, Collins, Bijlsma, et al., 2013). It is important to note that few studies have reported the frequency of IAs and the prevalence of HD in large cohorts of admixed populations worldwide (Agostinho et al., 2015; Apolinário, Paiva, & Agostinho, 2017; Kay et al., 2018; RASKIN et al., 2000). A study by Kay et al. (2018) investigated the epidemiology of HD and IAs in 15 populations with different ethnic origins but did not analyze the situation in Brazil in relation to intermediate HD alleles, which was the aim of our investigation.

Some of our results, such as the finding that 7.23% of individuals bore IAs in a sample of 235 individuals (470 chromosomes) from Rio de Janeiro, may be different from those of Raskin et al. (2000), as Brazil is a country of continental size, and the population of Paraná may differ from that of Rio de Janeiro concerning its ancestral origin; furthermore, the size of our sample was larger than theirs (235 vs. 50 subjects). For IA carriers, the risk of the transmission of an expanded allele generating new HD cases increases when the carriers harbor more than 30 CAG repeats (Semaka, Kay, Doty, Collins, Tam, et al., 2013; Semaka, Kay, Doty, Collins, Bijlsma, et al., 2013). The greater the number of CAGs, the greater the risk of the transmission of a more expanded allele to the next generation (Nahhas, Garbern, Krajewski, Roa, & Feldman, 2005; Warby et al., 2009; Wheeler et al., 2007). Kay et al. (2018) observed that the frequencies of IAs and large normal alleles were closely related to the prevalence of HD within the population (Kay et al., 2018), since it has been suggested that HD arises from a step‐by‐step mechanism in which during intergenerational transmission, large normal alleles can generate IAs, which are then responsible for generating de novo HD mutations (Warby et al., 2009). The same authors (Kay et al., 2018) analyzed the transmission of IAs from parents of European origin and concluded that there would be a probability of one de novo HD case in 3,260 births with ≥36 CAG repeats (Kay et al., 2018).

In our investigation, one individual was found to carry a reduced penetrance CAG allele with 37 repeats. In addition to presenting symptoms at some time in their lives, carriers of these alleles exhibit a high probability of transmitting a fully penetrant allele to their offspring in some cases (Kay et al., 2016; Nahhas et al., 2005). In the present study, the statistical analysis showed no significant difference between the frequencies of IAs in the two samples (GP and AS). A similar result was found in a study by Warby and colleagues involving individuals of European origin (Warby et al., 2009).

The mean numbers of CAG repeats among the normal chromosomes of the GP and AS were similar to the mean number of CAG repeats found among the normal chromosomes of the European population, which exhibits the highest prevalence of HD (Semaka, Kay, Doty, Collins, Tam, et al., 2013; Semaka, Kay, Doty, Collins, Bijlsma, et al., 2013; Squitieri et al., 1994).

When the normal CAG alleles of individuals from the GP were compared with the normal CAG alleles of individuals bearing another expanded allele (AS), no statistically significant difference was observed. This comparison between normal CAG alleles was performed because there are reports showing that trans factors may act by increasing the instability of the CAG repeats in a normal chromosome (Semaka, Kay, Doty, Collins, Tam, et al., 2013; Semaka, Kay, Doty, Collins, Bijlsma, et al., 2013).

When CCG alleles that segregated with normal CAG alleles and CCG alleles that segregated with expanded HD alleles were compared, a significant difference was identified between the groups. The most common CCG alleles in all studied populations worldwide exhibit 7 (CCG7) or 10 (CCG10) repeats. In populations with a higher prevalence of HD, such as that of Europe, the most frequently observed CCG allele presents seven repeats. In populations with a lower incidence of HD, such as those of Asian countries, the most prevalent allele is CCG10. It is believed that HD arose in Western Europe and that mutational events led to different numbers of CCG repeats (Hecimovic et al., 2002; Morovvati, Nakagawa, Osame, & Karami, 2008).

Our search showed that CCG7 alleles were the most commonly found alleles in chromosomes with normal CAG alleles as well as in those with IAs with reduced penetrance or full penetrance expanded alleles. However, CCG10 was more frequent in normal chromosomes than in expanded chromosomes in the AS. The most frequent CAG‐CCG haplotype per normal CAG allele was (CAG)17‐(CCG)7, and the most frequent haplotype per intermediate CAG allele was (CAG)27‐(CCG)7 in the GP. It is worth mentioning that these haplotypes were also the most frequent in the AS (Tables S2 and S4). To our knowledge, this is the first study to investigate CAG in phase with CCG (CAG/CCG haplotypes) in the GP residing in Rio de Janeiro city.

It is believed that the founding mutation for HD occurred in Western Europe and spread to other regions as a result of migration. Furthermore, the CCG7 allele is the predominant allele in Western Europe and could generate variations in the number of CCG repeats via independent mutational events (Hayden, Berkowicz, Beighton, & Yiptong, 1981). Since Brazil is a country of continental size with an admixed population, the determination of haplotypes A1‐A5, B, and C, which define haplogroups, must be performed for carriers of normal, intermediate, and expanded CAG alleles (Apolinário et al., 2017) in different subpopulations to obtain a better understanding of the ancestral origin of *HTT* mutations (Agostinho et al., 2012; 2015; Kay et al., 2019). Furthermore, genotyping with the identification of different haplotypes and the determination of haplogroups will be useful for achieving the allele‐specific silencing of *HTT* in HD‐affected individuals, in addition to investigating the hypothesis that haplotypes A1 and A2 are responsible for CAG tract instability. It is worth noting that early studies determined that the A1 and A2 haplotypes are most commonly associated with CAG tract expansion as well as with a greater average tract length in unexpanded alleles and are absent in low‐prevalence populations (Warby et al., 2011; 2012). These findings led to the theory that the A1 and A2 haplotypes themselves contribute to CAG tract instability and the risk of expansion (Warby et al., 2009, 2011). However, another hypothesis has been proposed: it has been postulated that the A1 and A2 haplotypes themselves are only bystanders in expansion, meaning that the founder haplotype is linked to a longer‐than‐average CAG tract by mere chance. Over many generations, considering the tendency of longer tracts to present higher instability and expansion rates, the linkage between these haplotypes and CAG tract expansion would have arisen (Falush et al., 2009). Through sperm cell analysis, Semaka, Kay, Doty, Collins, Tam et al. (2013) and Semaka, Kay, Doty, Collins, Bijlsma et al. (2013) demonstrated the instability of IA transmission to offspring. They showed that paternal germline CAG size‐specific risk estimates for IA instability increase the accuracy of clinical risk assessment for new mutations and provide data to help individuals make informed reproductive decisions.

It is important to mention that our results are not representative of the overall Brazilian population. As previously mentioned, Brazil is a country of continental size with different admixed populations with different ancestral origins (Europeans, African, and Amerindians); each of the 27 Brazilian states has a different ethnic composition based on different immigration fluxes over the years. Therefore, a sample of students from one specific academic institution in Rio de Janeiro (our GP) is not representative of the overall Brazilian population but is only a sample of the GP of Rio de Janeiro city. Furthermore, we emphasize that UNIRIO is a free State University that receives students of all socioeconomic and ethnic backgrounds, leading to a mixed sample.

## 5 CONCLUSION

The genetic profiles of the IAs obtained from the GP and the AS are rather similar. It is important to investigate the frequencies of IAs because expansions arise from a step‐by‐step mechanism in which, during intergenerational transmission, large normal alleles can generate IAs, which are then responsible for generating de novo HD mutations. The genetic investigation of IAs in GP was also important because it was focused on the population of Rio de Janeiro, Brazil, an understudied group. CCG7 was the most frequent CCG allele in linkage disequilibrium with normal, intermediate, and expanded CAG alleles, similar to the Western Europe population. However, a more robust investigation, in conjunction with haplogroup determination (A, B or C), will be required to elucidate the ancestral origin of the HTT in Brazilians.

## CONFLICT OF INTEREST

The authors have no conflict of interest to disclose.
