# Supplementary material for: Investigation of intermediate CAG alleles of the HTT in the general population of Rio de Janeiro, Brazil, in comparison with a sample of Huntington disease‐affected families
Source: Mol Genet Genomic Med. 2020 Feb 17;8(4):e1181. doi: 10.1002/mgg3.1181 (PMC7196456; doi:10.1002/mgg3.1181)
Supplement: Supplementary file 1 [file MGG3-8-e1181-s001.docx]

Supplementary tables

Table 1: CAG/CCG haplotypes of 259 individuals (518 chromosomes) **of the general population of Rio de Janeiro City**

| **Normal alleles (N=484)** | | |
| --- | --- | --- |
| **Numberofsample** | **Minor CAG allele**  **Haplotype (CAG) - (CCG)** | **Major CAG alelle**  **Haplotype (CAG)-(CCG)** |
|  | (CAG) 17- (CCG)7 | (CAG) 17- (CCG)10 |
|  | (CAG) 9- (CCG)9 | (CAG) 19- (CCG)7 |
|  | (CAG) 15- (CCG)9 | (CAG) 17- (CCG)8 |
|  | (CAG) 17- (CCG)7 | (CAG) 21- (CCG)7 |
|  | (CAG) 17- (CCG)7 | (CAG) 17- (CCG)7 |
|  | (CAG) 17- (CCG)7 | (CAG) 17- (CCG)7 |
|  | (CAG) 18- (CCG)10 | (CAG) 25- (CCG)7 |
|  | (CAG) 17- (CCG)10 | (CAG) 18- (CCG)10 |
|  | (CAG) 16- (CCG)7 | (CAG) 18- (CCG)10 |
|  | (CAG) 19- (CCG)7 | (CAG) 23- (CCG)7 |
|  | (CAG) 9- (CCG)8 | (CAG) 19- (CCG)7 |
|  | (CAG) 17- (CCG)10 | (CAG) 18- (CCG)10 |
|  | (CAG) 18- (CCG)7 | (CAG) 19- (CCG)7 |
|  | (CAG) 18- (CCG)7 | (CAG) 24- (CCG)7 |
|  | (CAG) 18- (CCG)7 | (CAG) 22- (CCG)7 |
|  | (CAG) 18- (CCG)7 | (CAG) 21- (CCG)7 |
|  | (CAG) 17- (CCG)7 | (CAG) 24- (CCG)7 |
|  | (CAG) 17- (CCG)8 | (CAG) 18- (CCG)9 |
|  | (CAG) 17- (CCG)7 | (CAG) 18- (CCG)10 |
|  | (CAG) 17- (CCG)7 | (CAG) 19- (CCG)7 |
|  | (CAG) 16- (CCG)7 | (CAG) 18- (CCG)7 |
|  | (CAG) 18- (CCG)7 | (CAG) 26- (CCG)7 |
|  | (CAG) 17- (CCG)7 | (CAG) 18- (CCG)10 |
|  | (CAG) 16- (CCG)7 | (CAG) 19- (CCG)7 |
|  | (CAG) 15- (CCG)7 | (CAG) 20- (CCG)10 |
|  | (CAG) 15- (CCG)7 | (CAG) 17- (CCG)7 |
|  | (CAG) 9- (CCG)10 | (CAG) 17- (CCG)7 |
|  | (CAG) 16- (CCG)7 | (CAG) 17- (CCG)7 |
|  | (CAG) 10- (CCG)10 | (CAG) 23- (CCG)7 |
|  | (CAG) 17- (CCG)8 | (CAG) 22- (CCG)7 |
|  | (CAG) 15- (CCG)10 | (CAG) 17- (CCG)7 |
|  | (CAG) 15- (CCG)7 | (CAG) 17- (CCG)10 |
|  | (CAG) 17- (CCG)10 | (CAG) 17- (CCG)7 |
|  | (CAG) 17- (CCG)10 | (CAG) 24- (CCG)7 |
|  | (CAG) 19- (CCG)7 | (CAG) 26- (CCG)10 |
|  | (CAG) 16- (CCG)7 | (CAG) 18- (CCG)9 |
|  | (CAG) 17- (CCG)7 | (CAG) 26- (CCG)7 |
|  | (CAG) 17- (CCG)7 | (CAG) 21- (CCG)7 |
|  | (CAG) 17- (CCG)7 | (CAG) 17- (CCG)7 |
|  | (CAG) 17- (CCG)7 | (CAG) 17- (CCG)7 |
|  | (CAG) 13- (CCG)7 | (CAG) 16- (CCG)6 |
|  | (CAG) 18- (CCG)7 | (CAG) 22- (CCG)7 |
|  | (CAG) 16- (CCG)7 | (CAG) 19- (CCG)10 |
|  | (CAG) 17- (CCG)10 | (CAG) 23- (CCG)7 |
|  | (CAG) 17- (CCG)9 | (CAG) 18- (CCG)7 |
|  | (CAG) 17- (CCG)7 | (CAG) 17- (CCG)9 |
|  | (CAG) 15- (CCG)10 | (CAG) 18- (CCG)9 |
|  | (CAG) 17- (CCG)7 | (CAG) 21- (CCG)7 |
|  | (CAG) 16- (CCG)7 | (CAG) 18- (CCG)10 |
|  | (CAG) 17- (CCG)7 | (CAG) 18- (CCG)10 |
|  | (CAG) 18- (CCG)7 | (CAG) 18- (CCG)10 |
|  | (CAG) 17- (CCG)7 | (CAG) 18- (CCG)10 |
|  | (CAG) 17- (CCG)7 | (CAG) 18- (CCG)7 |
|  | (CAG) 19- (CCG)10 | (CAG) 21- (CCG)7 |
|  | (CAG) 15- (CCG)10 | (CAG) 17- (CCG)7 |
|  | (CAG) 18- (CCG)10 | (CAG) 18- (CCG)10 |
|  | (CAG) 17- (CCG)7 | (CAG) 26- (CCG)7 |
|  | (CAG) 15- (CCG)10 | (CAG) 17- (CCG)7 |
|  | (CAG) 15- (CCG)10 | (CAG) 20- (CCG)7 |
|  | (CAG) 17- (CCG)7 | (CAG) 22- (CCG)7 |
|  | (CAG) 20- (CCG)7 | (CAG) 20- (CCG)10 |
|  | (CAG) 17- (CCG)X | (CAG) 17- (CCG)X |
|  | (CAG) 17- (CCG)7 | (CAG) 19- (CCG)10 |
|  | (CAG) 16- (CCG)7 | (CAG) 18- (CCG)7 |
|  | (CAG) 10- (CCG) 10 | (CAG) 17- (CCG)7 |
|  | (CAG) 16- (CCG)10 | (CAG) 18- (CCG)10 |
|  | (CAG) 16- (CCG)7 | (CAG) 16- (CCG)7 |
|  | (CAG) 17- (CCG)10 | (CAG) 21- (CCG)7 |
|  | (CAG) 16- (CCG)7 | (CAG) 28- (CCG)7 |
|  | (CAG) 17- (CCG)10 | (CAG) 24- (CCG)7 |
|  | (CAG) 11- (CCG)10 | (CAG) 22- (CCG)10 |
|  | (CAG) 17- (CCG)10 | (CAG) 18- (CCG)10 |
|  | (CAG) 17- (CCG)10 | (CAG) 20- (CCG)7 |
|  | (CAG) 15- (CCG)7 | (CAG) 26- (CCG)7 |
|  | (CAG) 17- (CCG)7 | (CAG) 22- (CCG)7 |
|  | (CAG) 18- (CCG)10 | (CAG) 25- (CCG)7 |
|  | (CAG) 16- (CCG)7 | (CAG) 20- (CCG)7 |
|  | (CAG) 13- (CCG)7 | (CAG) 18- (CCG)7 |
|  | (CAG) 19- (CCG)7 | (CAG) 19- (CCG)10 |
|  | (CAG) 17- (CCG)10 | (CAG) 18- (CCG)10 |
|  | (CAG) 17- (CCG)7 | (CAG) 17- (CCG)10 |
|  | (CAG) 16- (CCG)10 | (CAG) 18- (CCG)7 |
|  | (CAG) 17 (CCG)10 | (CAG) 23- (CCG)7 |
|  | (CAG) 15- (CCG)9 | (CAG) 17- (CCG)7 |
|  | (CAG) 17- (CCG)7 | (CAG) 18- (CCG)10 |
|  | (CAG) 16- (CCG)6 | (CAG) 19- (CCG)7 |
|  | (CAG) 17- (CCG)7 | (CAG) 17- (CCG)7 |
|  | (CAG) 16- (CCG)7 | (CAG) 23- (CCG)7 |
|  | (CAG) 16- (CCG)7 | (CAG) 17- (CCG)8 |
|  | (CAG) 15- (CCG)7 | (CAG) 18- (CCG)7 |
|  | (CAG) 15- (CCG)X | (CAG) 17- (CCG)X |
|  | (CAG) 17- (CCG)7 | (CAG) 17- (CCG)10 |
|  | (CAG) 17- (CCG)7 | (CAG) 19- (CCG)7 |
|  | (CAG) 15- (CCG)10 | (CAG) 17- (CCG)7 |
|  | (CAG) 17- (CCG)7 | (CAG) 19- (CCG)9 |
|  | (CAG) 16- (CCG)X | (CAG) 17- (CCG)X |
|  | (CAG) 11- (CCG)10 | (CAG) 15- (CCG)10 |
|  | (CAG) 17- (CCG)7 | (CAG) 19- (CCG)10 |
|  | (CAG) 18- (CCG)X | (CAG) 19- (CCG)X |
|  | (CAG) 17- (CCG)7 | (CAG) 21- (CCG)7 |
|  | (CAG) 17- (CCG)7 | (CAG) 25- (CCG)7 |
|  | (CAG) 21- (CCG)7 | (CAG) 26- (CCG)10 |
|  | (CAG) 17- (CCG)10 | (CAG) 18- (CCG)10 |
|  | (CAG) 17- (CCG)7 | (CAG) 18- (CCG)10 |
|  | (CAG) 17- (CCG)7 | (CAG) 17- (CCG)10 |
|  | (CAG) 15- (CCG)7 | (CAG) 19- (CCG)10 |
|  | (CAG) 18- (CCG)7 | (CAG) 18- (CCG)7 |
|  | (CAG) 18- (CCG)7 | (CAG) 19- (CCG)7 |
|  | (CAG) 16- (CCG)7 | (CAG) 17- (CCG)7 |
|  | (CAG) 19- (CCG)7 | (CAG) 23- (CCG)7 |
|  | (CAG) 18- (CCG)7 | (CAG) 18- (CCG)7 |
|  | (CAG) 18- (CCG)7 | (CAG) 18- (CCG)9 |
|  | (CAG) 16- (CCG)X | (CAG) 17- (CCG)X |
|  | (CAG) 15- (CCG)10 | (CAG) 25- (CCG)7 |
|  | (CAG) 15- (CCG)7 | (CAG) 17- (CCG)10 |
|  | (CAG) 17- (CCG)5 | (CAG) 17- (CCG)10 |
|  | (CAG) 16- (CCG)7 | (CAG) 19- (CCG)10 |
|  | (CAG) 15- (CCG)10 | (CAG) 17- (CCG)7 |
|  | (CAG) 17- (CCG)7 | (CAG) 20- (CCG)7 |
|  | (CAG) 18- (CCG)7 | (CAG) 22- (CCG)7 |
|  | (CAG) 17- (CCG)10 | (CAG) 18- (CCG)7 |
|  | (CAG) 18- (CCG)7 | (CAG) 18- (CCG)10 |
|  | (CAG) 15- (CCG)7 | (CAG) 18- (CCG)7 |
|  | (CAG) 15- (CCG)10 | (CAG) 18- (CCG)7 |
|  | (CAG) 15- (CCG)7 | (CAG) 23- (CCG)7 |
|  | (CAG) 18- (CCG)7 | (CAG) 19- (CCG)7 |
|  | (CAG) 17- (CCG)7 | (CAG) 17- (CCG)7 |
|  | (CAG) 17- (CCG)10 | (CAG) 18- (CCG)10 |
|  | (CAG) 15- (CCG)10 | (CAG) 19- (CCG)10 |
|  | (CAG) 16- (CCG)7 | (CAG) 17- (CCG)8 |
|  | (CAG) 17- (CCG)7 | (CAG) 17- (CCG)7 |
|  | (CAG) 16- (CCG)7 | (CAG) 24- (CCG)7 |
|  | (CAG) 17- (CCG)10 | (CAG) 19- (CCG)7 |
|  | (CAG) 16- (CCG)7 | (CAG) 16- (CCG)10 |
|  | (CAG) 17- (CCG)7 | (CAG) 17- (CCG)10 |
|  | (CAG) 16- (CCG)7 | (CAG) 18- (CCG)10 |
|  | (CAG) 17- (CCG)10 | (CAG) 18- (CCG)7 |
|  | (CAG) 17- (CCG)10 | (CAG) 20- (CCG)7 |
|  | (CAG) 18- (CCG)10 | (CAG) 19- (CCG)7 |
|  | (CAG) 16- (CCG)7 | (CAG) 17- (CCG)7 |
|  | (CAG) 18- (CCG)7 | (CAG) 19- (CCG)7 |
|  | (CAG) 15- (CCG)7 | (CAG) 24- (CCG)7 |
|  | (CAG) 17- (CCG)7 | (CAG) 17- (CCG)7 |
|  | (CAG) 17- (CCG)7 | (CAG) 17- (CCG)7 |
|  | (CAG) 15- (CCG)7 | (CAG) 22- (CCG)7 |
|  | (CAG) 17- (CCG)6 | (CAG) 17- (CCG)7 |
|  | (CAG) 17- (CCG)7 | (CAG) 17- (CCG)7 |
|  | (CAG) 17- (CCG)10 | (CAG) 19- (CCG)8 |
|  | (CAG) 15- (CCG)10 | (CAG) 18- (CCG)7 |
|  | (CAG) 18- (CCG)7 | (CAG) 20- (CCG)7 |
|  | (CAG) 15- (CCG)7 | (CAG) 17- (CCG)7 |
|  | (CAG) 18- (CCG)7 | (CAG) 24- (CCG)7 |
|  | (CAG) 16- (CCG)10 | (CAG) 17- (CCG)7 |
|  | (CAG) 17- (CCG)7 | (CAG) 21- (CCG)7 |
|  | (CAG) 17- (CCG)10 | (CAG) 20- (CCG)7 |
|  | (CAG) 16- (CCG)7 | (CAG) 18- (CCG)10 |
|  | (CAG) 17- (CCG)10 | (CAG) 17- (CCG)10 |
|  | (CAG) 10- (CCG)10 | (CAG) 16- (CCG)10 |
|  | (CAG) 24- (CCG)7 | (CAG) 26- (CCG)7 |
|  | (CAG) 18- (CCG)7 | (CAG) 21- (CCG)7 |
|  | (CAG) 15- (CCG)10 | (CAG) 15- (CCG)10 |
|  | (CAG) 15- (CCG)10 | (CAG) 18- (CCG)10 |
|  | (CAG) 17- (CCG)7 | (CAG) 19- (CCG)7 |
|  | (CAG) 19- (CCG)7 | (CAG) 19- (CCG)7 |
|  | (CAG) 20- (CCG)7 | (CAG) 20- (CCG)7 |
|  | (CAG) 17- (CCG)7 | (CAG) 21- (CCG)7 |
|  | (CAG) 15- (CCG)7 | (CAG) 15- (CCG)10 |
|  | (CAG) 17- (CCG)7 | (CAG) 17- (CCG)7 |
|  | (CAG) 15- (CCG)10 | (CAG) 16- (CCG)9 |
|  | (CAG) 16- (CCG)7 | (CAG) 20- (CCG)10 |
|  | (CAG) 19- (CCG)10 | (CAG) 25- (CCG)7 |
|  | (CAG) 16- (CCG)7 | (CAG) 18- (CCG)10 |
|  | (CAG) 17- (CCG)7 | (CAG) 23- (CCG)7 |
|  | (CAG) 17- (CCG)10 | (CAG) 19- (CCG)8 |
|  | (CAG) 17- (CCG)10 | (CAG) 20- (CCG)7 |
|  | (CAG) 16- (CCG)7 | (CAG) 17- (CCG)8 |
|  | (CAG) 15- (CCG)7 | (CAG) 17- (CCG)7 |
|  | (CAG) 21- (CCG)7 | (CAG) 18- (CCG)10 |
|  | (CAG) 18- (CCG)7 | (CAG) 27- (CCG)7 |
|  | (CAG) 17- (CCG)7 | (CAG) 25- (CCG)7 |
|  | (CAG) 17- (CCG)7 | (CAG) 20- (CCG)7 |
|  | (CAG) 17- (CCG)10 | (CAG) 26- (CCG)7 |
|  | (CAG) 17- (CCG)7 | (CAG) 17- (CCG)10 |
|  | (CAG) 15- (CCG)10 | (CAG) 17- (CCG)8 |
|  | (CAG) 17- (CCG)7 | (CAG) 21- (CCG)10 |
|  | (CAG) 17- (CCG)7 | (CAG) 17- (CCG)7 |
|  | (CAG) 17- (CCG)8 | (CAG) 17- (CCG)8 |
|  | (CAG) 19- (CCG)10 | (CAG) 25- (CCG)7 |
|  | (CAG) 18- (CCG)X | (CAG) 22- (CCG)X |
|  | (CAG) 16- (CCG)7 | (CAG) 23- (CCG)7 |
|  | (CAG) 17- (CCG)10 | (CAG) 23- (CCG)7 |
|  | (CAG) 15- (CCG)7 | (CAG) 17- (CCG)10 |
|  | (CAG) 15- (CCG)10 | (CAG) 16- (CCG)9 |
|  | (CAG) 17- (CCG)7 | (CAG) X- (CCG)10 |
|  | (CAG) 16- (CCG)7 | (CAG) 24- (CCG)7 |
|  | (CAG) 17- (CCG)7 | (CAG) 18- (CCG)10 |
|  | (CAG) 15- (CCG)7 | (CAG) X- (CCG)10 |
|  | (CAG) 14- (CCG)7 | (CAG) 15- (CCG)10 |
|  | (CAG) 17- (CCG)7 | (CAG) 18- (CCG)7 |
|  | (CAG) 16- (CCG)7 | (CAG) 19- (CCG)10 |
|  | (CAG) 16- (CCG)7 | (CAG) 17- (CCG)7 |
|  | (CAG) 17- (CCG)10 | (CAG) 18- (CCG)7 |
|  | (CAG) 17- (CCG)X | (CAG) 18- (CCG)X |
|  | (CAG) 15- (CCG)10 | (CAG) 17- (CCG)10 |
|  | (CAG) 17- (CCG)7 | (CAG) 17- (CCG)9 |
|  | (CAG) 17- (CCG)7 | (CAG) 17- (CCG)7 |
|  | (CAG) 17- (CCG)7 | (CAG) 18- (CCG)7 |
|  | (CAG) 16- (CCG)7 | (CAG) 18- (CCG)10 |
|  | (CAG) 20- (CCG)7 | (CAG) 23- (CCG)7 |
|  | (CAG) 16- (CCG)7 | (CAG) 17- (CCG)8 |
|  | (CAG) 18- (CCG)10 | (CAG) 19- (CCG)10 |
|  | (CAG) 17- (CCG)6 | (CAG) 17- (CCG)6 |
|  | (CAG) 18- (CCG)7 | (CAG) 19- (CCG)7 |
|  | (CAG) 15- (CCG)7 | (CAG) 19- (CCG)7 |
|  | (CAG) 18- (CCG)10 | (CAG) 18- (CCG)10 |
|  | (CAG) 17- (CCG)7 | (CAG) 17- (CCG)10 |
|  | (CAG) 16- (CCG)7 | (CAG) 19- (CCG)10 |
|  | (CAG) 17- (CCG)7 | (CAG) 18- (CCG)7 |
|  | (CAG) 17- (CCG)7 | (CAG) 19- (CCG)7 |
|  | (CAG) 17- (CCG)10 | (CAG) 19- (CCG)10 |
|  | (CAG) 17- (CCG)7 | (CAG) 23- (CCG)10 |
|  | (CAG) 17- (CCG)10 | (CAG) 23- (CCG)7 |
|  | (CAG) 18- (CCG)10 | (CAG) 26- (CCG)7 |
|  | (CAG) 15- (CCG)9 | (CAG) 17- (CCG)7 |
|  | (CAG) 15- (CCG)10 | (CAG) 19- (CCG)10 |
|  | (CAG) 17- (CCG)7 | (CAG) 18- (CCG)10 |
|  | (CAG) 19- (CCG)10 | (CAG) 25- (CCG)7 |
|  | (CAG) 15- (CCG)7 | (CAG) 19- (CCG)7 |
|  | (CAG) 15- (CCG)9 | (CAG) 17- (CCG)7 |
|  | (CAG) 20- (CCG)7 | (CAG) 24- (CCG)7 |
|  | (CAG) 15- (CCG)10 | (CAG) 18- (CCG)10 |
|  | (CAG) 17- (CCG)10 | (CAG) 20- (CCG)10 |
|  | (CAG) 17- (CCG)7 | (CAG) 17- (CCG)10 |
|  | (CAG) 15- (CCG)9 | (CAG) 17- (CCG)7 |
|  | (CAG) 17- (CCG)7 | (CAG) 17- (CCG)7 |
|  | (CAG) 15- (CCG)7 | (CAG) 15- (CCG)7 |
|  | (CAG) 15- (CCG)10 | (CAG) 18- (CCG)10 |
|  | (CAG) 17- (CCG)10 | (CAG) 18- (CCG)10 |
|  | (CAG) 15- (CCG)7 | (CAG) 17- (CCG)9 |
|  | (CAG) 19- (CCG)7 | (CAG) 21- (CCG)7 |
|  | (CAG) 18- (CCG)7 | (CAG) 20- (CCG)10 |
|  | (CAG) 17- (CCG)7 | (CAG) 17- (CCG)7 |
| 264. | (CAG) 17- (CCG)7 | (CAG) 17- (CCG)9 |
| **Intermediate alleles** | | |
| 46. | (CAG) 15- (CCG)10 | (CAG) 27- (CCG)7 |
| 59. | (CAG) 21- (CCG)10 | (CAG) 27- (CCG)7 |
| 62. | (CAG) 18- (CCG)7 | (CAG) 28- (CCG)7 |
| 66. | (CAG) 19- (CCG)7 | (CAG) 30- (CCG)7 |
| 76. | (CAG) 20- (CCG)10 | (CAG) 28- (CCG)7 |
| 107. | (CAG) 17- (CCG)7 | (CAG) 31- (CCG)7 |
| 123. | (CAG) 16- (CCG)6 | (CAG) 30- (CCG)6 |
| 130. | (CAG) 15- (CCG)10 | (CAG) 27- (CCG)7 |
| 145. | (CAG) 10- (CCG)10 | (CAG) 27- (CCG)7 |
| 161. | (CAG) 15- (CCG)10 | (CAG) 27- (CCG)7 |
| 226. | (CAG) 20- (CCG)7 | (CAG) 29- (CCG)7 |
| 227. | (CAG) 20- (CCG)10 | (CAG) 31- (CCG)10 |
| 229. | (CAG) 19- (CCG)7 | (CAG) 28- (CCG)7 |
| 253. | (CAG) 17- (CCG)9 | (CAG) 30- (CCG)10 |
| 254. | (CAG) 15- (CCG)10 | (CAG) 31- (CCG)7 |
| **Reduced penetrance allele (N=1)** | | |
| 208 | (CAG) 15- (CCG)10 | (CAG) 37- (CCG)7 |

Legend: (CCG) X = CCG allele not determined.

Table 2: Frequency of CAG/CCG haplotypes (n=518 chromosomes)

| **CAG-CCG Haplotype** | **Number of haplotypes** | **Frequency** |
| --- | --- | --- |
| **Normal alleles (N=484)** | | |
| (CAG) 9- (CCG)9 | 1 | 0,002 |
| **(CAG) 17- (CCG)7** | **101** | **0,208** |
| (CAG) 15- (CCG)9 | 5 | 0,010 |
| (CAG) 18- (CCG)10 | 37 | 0,076 |
| (CAG) 17- (CCG)10 | 42 | 0,086 |
| (CAG) 16- (CCG)7 | 32 | 0,066 |
| (CAG) 18- (CCG)7 | 40 | 0,082 |
| (CAG) 19- (CCG)10 | 16 | 0,033 |
| (CAG) 15- (CCG)7 | 21 | 0,043 |
| (CAG) 15- (CCG)10 | 29 | 0,059 |
| (CAG) 19- (CCG)7 | 26 | 0,053 |
| (CAG) 9- (CCG)8 | 1 | 0,002 |
| (CAG) 17- (CCG)8 | 10 | 0,020 |
| (CAG) 9- (CCG)10 | 1 | 0,002 |
| (CAG) 10- (CCG)10 | 4 | 0,008 |
| (CAG) 13- (CCG)7 | 2 | 0,004 |
| (CAG) 17- (CCG)9 | 6 | 0,012 |
| (CAG) 21- (CCG)10 | 2 | 0,004 |
| (CAG) 20- (CCG)7 | 15 | 0,030 |
| (CAG) 16- (CCG)10 | 5 | 0,010 |
| (CAG) 20- (CCG)10 | 7 | 0,014 |
| (CAG) 11- (CCG)10 | 2 | 0,004 |
| (CAG) 17 (CCG)10 | 1 | 0,002 |
| (CAG) 16- (CCG)6 | 3 | 0,006 |
| (CAG) 21- (CCG)7 | 13 | 0,026 |
| (CAG) 17- (CCG)5 | 1 | 0,002 |
| (CAG) 17- (CCG)6 | 3 | 0,006 |
| (CAG) 24- (CCG)7 | 10 | 0,020 |
| (CAG) 14- (CCG)7 | 1 | 0,002 |
| (CAG) 25- (CCG)7 | 8 | 0,016 |
| (CAG) 23- (CCG)7 | 12 | 0,024 |
| (CAG) 22- (CCG)7 | 7 | 0,014 |
| (CAG) 18- (CCG)9 | 4 | 0,008 |
| (CAG) 26- (CCG)7 | 7 | 0,014 |
| (CAG) 26- (CCG)10 | 2 | 0,004 |
| (CAG) 22- (CCG)10 | 1 | 0,002 |
| (CAG) 19- (CCG)9 | 1 | 0,002 |
| (CAG) 19- (CCG)8 | 2 | 0,004 |
| (CAG) 16- (CCG)9 | 2 | 0,004 |
| (CAG) 23- (CCG)10 | 1 | 0,002 |
|  | | |
| **Intermediate alleles (n=17)** | | |
| **(CAG) 27- (CCG)7** | **6** | **0,352** |
| (CAG) 28- (CCG)7 | 4 | 0,008 |
| (CAG) 30- (CCG)7 | 1 | 0,058 |
| (CAG) 31- (CCG)7 | 2 | 0,117 |
| (CAG) 30- (CCG)6 | 1 | 0,058 |
| (CAG) 29- (CCG)7 | 1 | 0,058 |
| (CAG) 31- (CCG)10 | 1 | 0,058 |
| (CAG) 30- (CCG)10 | 1 | 0,058 |
|  | | |
| **Reducedpenetranceallele (N= 1)** | | |
| **(CAG) 37- (CCG)7** | **1** | **1** |

Table 3: Phased CAG /CCG repeats of 55 individuals **from affected families** (n=110 chromosomes)

| **Samplenumber** | **Minor CAG alelle**  **Haplotype (CAG) - (CCG)** | **Major CAG alelle**  **Haplotype (CAG)-(CCG)** |
| --- | --- | --- |
| 1. | (CAG)23--(CCG)7 | (CAG)25--(CCG)7 |
| 6. | (CAG)17--(CCG)7 | (CAG)17--(CCG)7 |
| 17. | (CAG)17--(CCG)10 | (CAG)18--(CCG)10 |
| 22. | (CAG)18--(CCG)8 | (CAG)19--(CCG)10 |
| 56. | (CAG)17--(CCG)7 | (CAG)19--(CCG)7 |
| 70. | (CAG)17--(CCG)10 | (CAG)18--(CCG)10 |
| 77. | (CAG)14--(CCG)7 | (CAG)21--(CCG)10 |
| 91. | (CAG)15--(CCG)9 | (CAG)25--(CCG)7 |
| 94. | (CAG)15--(CCG)7 | (CAG)19--(CCG)7 |
| 95. | (CAG)15--(CCG)10 | (CAG)17--(CCG)7 |
| 107. | (CAG)17--(CCG)7 | (CAG)19--(CCG)7 |
| 112. | (CAG)16--(CCG)6 | (CAG)20--(CCG)7 |
| 113. | (CAG)17--(CCG)7 | (CAG)20--(CCG)7 |
| 114. | (CAG)16--(CCG)6 | (CAG)19--(CCG)10 |
| 117. | (CAG)17--(CCG)7 | (CAG)17--(CCG)7 |
| 118. | (CAG)15--(CCG)10 | (CAG)20--(CCG)7 |
| 121. | (CAG)17--(CCG)8 | (CAG)17--(CCG)7 |
| 126. | (CAG)17--(CCG)7 | (CAG)19--(CCG)7 |
| **Intermediate CAG alleles/CCG segregating with normal alleles** | | |
| 72. | (CAG)17--(CCG)7 | (CAG)27--(CCG)7 |
| **Intermediate alleles segregating with expanded alleles** | | |
| 9. | (CAG)27--(CCG)7 | (CAG)45--(CCG)7 |
| 52. | (CAG)29--(CCG)7 | (CAG)45--(CCG)7 |
| 64. | (CAG)28--(CCG)7 | (CAG)43--(CCG)7 |
| **Expanded alelles** | | |
| 2. | (CAG)18--(CCG)10 | (CAG)46--(CCG)7 |
| 8. | (CAG)17--(CCG)7 | (CAG)42--(CCG)7 |
| 12. | (CAG)17--(CCG)10 | (CAG)43--(CCG)7 |
| 14. | (CAG)15--(CCG)7 | (CAG)46--(CCG)7 |
| 15. | (CAG)15--(CCG)7 | (CAG)46--(CCG)7 |
| 18. | (CAG)19--(CCG)10 | (CAG)44--(CCG)7 |
| 19. | (CAG)22--(CCG)7 | (CAG)62--(CCG)7 |
| 20. | (CAG)25--(CCG)7 | (CAG)45--(CCG)7 |
| 21. | (CAG)16--(CCG)7 | (CAG)51--(CCG)7 |
| 53. | (CAG)17--(CCG)7 | (CAG)49--(CCG)7 |
| 54. | (CAG)17--(CCG)10 | (CAG)43--(CCG)7 |
| 55. | (CAG)17--(CCG)7 | (CAG)42--(CCG)7 |
| 58. | (CAG)17--(CCG)10 | (CAG)43--(CCG)7 |
| 59. | (CAG)17--(CCG)10 | (CAG)42--(CCG)7 |
| 62. | (CAG)17--(CCG)7 | (CAG)40--(CCG)7 |
| 65. | (CAG)24--(CCG)7 | (CAG)48--(CCG)7 |
| 68. | (CAG)17--(CCG)7 | (CAG)41--(CCG)7 |
| 69. | (CAG)15--(CCG)7 | (CAG)44--(CCG)7 |
| 71. | (CAG)14--(CCG)10 | (CAG)52--(CCG)7 |
| 78. | (CAG)17--(CCG)7 | (CAG)44--(CCG)7 |
| 83. | (CAG)23--(CCG)10 | (CAG)44--(CCG)7 |
| 84. | (CAG)18--(CCG)10 | (CAG)46--(CCG)7 |
| 85. | (CAG)17--(CCG)7 | (CAG)41--(CCG)7 |
| 87. | (CAG)19--(CCG)7 | (CAG)41--(CCG)7 |
| 88. | (CAG)17--(CCG)7 | (CAG)48--(CCG)7 |
| 92. | (CAG)25--(CCG)7 | (CAG)45--(CCG)7 |
| 101. | (CAG)26--(CCG)7 | (CAG)40--(CCG)10 |
| 103. | (CAG)17--(CCG)7 | (CAG)45--(CCG)7 |
| 115. | (CAG)16--(CCG)7 | (CAG)51--(CCG)7 |
| 116. | (CAG)18--(CCG)X | (CAG)74--(CCG)X |
| 120. | (CAG)17--(CCG)10 | (CAG)47--(CCG)7 |
| 123. | (CAG)17--(CCG)7 | (CAG)45--(CCG)7 |
| 124. | (CAG)26--(CCG)10 | (CAG)56--(CCG)7 |

Legend: (CCG) X = CCG allele not determined.

Tabela 4: Phased CAG /CCG repeats of chromosomes **from affected families (n=110 chromosomes)**

| **CAG-CCG Haplotype** | **Numberofhaplotypes** | **Frequency** |
| --- | --- | --- |
| **Normal alleles (N=69)** | | |
| (CAG)14--(CCG)10 | 1 | 0,014 |
| (CAG)14--(CCG)7 | 1 | 0,014 |
| (CAG)15--(CCG)10 | 2 | 0,028 |
| (CAG)15--(CCG)7 | 4 | 0,057 |
| (CAG)15--(CCG)9 | 1 | 0,014 |
| (CAG)16--(CCG)6 | 2 | 0,028 |
| (CAG)16--(CCG)7 | 2 | 0,028 |
| (CAG)17--(CCG)10 | 7 | 0,101 |
| **(CAG)17--(CCG)7** | **21** | **0,304** |
| (CAG)17--(CCG)8 | 1 | 0,014 |
| (CAG)18--(CCG)10 | 4 | 0,057 |
| (CAG)18--(CCG)8 | 1 | 0,014 |
| (CAG)19--(CCG)10 | 3 | 0,043 |
| (CAG)19--(CCG)7 | 5 | 0,072 |
| (CAG)20--(CCG)7 | 3 | 0,043 |
| (CAG)21--(CCG)10 | 1 | 0,014 |
| (CAG)22--(CCG)7 | 1 | 0,014 |
| (CAG)23--(CCG)10 | 1 | 0,014 |
| (CAG)23--(CCG)7 | 1 | 0,014 |
| (CAG)24--(CCG)7 | 1 | 0,014 |
| (CAG)25--(CCG)7 | 4 | 0,057 |
| (CAG)26--(CCG)10 | 1 | 0,014 |
| (CAG)26--(CCG)7 | 1 | 0,014 |
|  | | |
| **Intermediate alleles (N= 4)** | | |
| **(CAG)27--(CCG)7** | **2** | **0,5** |
| (CAG)28--(CCG)7 | 1 | 0,25 |
| (CAG)29--(CCG)7 | 1 | 0,25 |
|  | | |
| **Expanded allele (N= 35)** | | |
| (CAG)40--(CCG)10 | 1 | 0,028 |
| (CAG)40--(CCG)7 | 1 | 0,028 |
| (CAG)41--(CCG)7 | 3 | 0,085 |
| (CAG)42--(CCG)7 | 3 | 0,085 |
| (CAG)43--(CCG)7 | 4 | 0,114 |
| (CAG)44--(CCG)7 | 4 | 0,114 |
| (CAG)45--(CCG)7 | 6 | 0,171 |
| (CAG)46--(CCG)7 | 4 | 0,114 |
| (CAG)47--(CCG)7 | 1 | 0,028 |
| (CAG)48--(CCG)7 | 2 | 0,057 |
| (CAG)49--(CCG)7 | 1 | 0,028 |
| (CAG)51--(CCG)7 | 2 | 0,057 |
| (CAG)52--(CCG)7 | 1 | 0,028 |
| (CAG)56--(CCG)7 | 1 | 0,028 |
| (CAG)62--(CCG)7 | 1 | 0,028 |

Table 5: Categorization of all classes of CAG alleles found in individuals of both groups: ener general population and affected sample.

|  | General population | | | | | Affected Sample | | | | |
| --- | --- | --- | --- | --- | --- | --- | --- | --- | --- | --- |
| **Category**  **CAG alleles** | **Mean** | **SD** | **Median** | **Absolute frequency** | **Relative frequency** | **Mean** | **SD** | **Median** | **Absolute frequency** | **Relative frequency** |
| Normal | 17.8 | ±2.6 | 17 | 452 | 96.2% | 18.2 | ±2.9 | 17 | 70 | 63.6% |
| Intermediate | 28.6 | ±1.6 | 28 | 17 | 3.6% | 27.7 | ±0.9 | 27 | 4 | 3.6% |
| Reduced penetrance | - | - | - | 1 | 0.2% | - | - | - | - | - |
| Full penetrance | - | - | - | - | - | 46.36 | ±6.5 | 45 | 36 | 32.7% |

Legend: SD= Standard deviation.
